# Exogenous gene transfer of Rab^3^8 small GTPase ameliorates aberrant lung surfactant homeostasis in *Ruby* rats

**DOI:** 10.1186/s12931-017-0549-2

**Published:** 2017-04-24

**Authors:** Kazuhiro Osanai, Keisuke Nakase, Takashi Sakuma, Kazuaki Nishiki, Masafumi Nojiri, Ryo Kato, Masatoshi Saito, Yuki Fujimoto, Shiro Mizuno, Hirohisa Toga

**Affiliations:** Department of Respiratory Medicine, Kanazawa Medical University, 1-1 Uchinada-Daigaku, Kahokugun, Ishikawa, 920-0293 Japan

**Keywords:** Rab^3^8 GTPase, *Ruby* rats, Gene transfer, Adenovector, Lung surfactant, Hermansky-Pudlak syndrome

## Abstract

**Background:**

Rab^3^8 small GTPase regulates intracellular transport in melanocytes and alveolar type II epithelial cells. *Ruby* rats carrying Rab^3^8 and other gene mutations exhibit oculocutaneous albinism, bleeding diathesis, and hence, are a rat model of human Hermansky-Pudlak syndrome (HPS). We previously showed that Long Evans Cinnamon (LEC) rats, one strain of the *Ruby* rats, developed aberrant lung surfactant homeostasis with remarkably enlarged lamellar bodies in alveolar type II cells.

**Methods:**

A replication-deficient recombinant adenovirus expressing rat Rab^3^8 (Ad-Rab^3^8) was constructed. Alveolar type II cells were isolated from the LEC rats and tested for lung surfactant phosphatidylcholine secretion. The rats were also examined whether exogenous expression of Ad- Rab^3^8 could rescue the altered lung surfactant homeostasis in the lungs.

**Results:**

Isolated type II cells infected with Ad-Rab^3^8 exhibited improved secretion patterns of [^3^H]phosphatidylcholine, i.e. increased basal hyposecretion and decreased agonist-induced hypersecretion. Endobronchial administration of Ad-Rab^3^8 improved the morphology of type II cells and lamellar bodies, reducing their sizes close to those of wild-type rats. The increased amounts of phosphatidylcholine and surfactant protein B in the lamellar body fractions were decreased in the Ad-Rab^3^8 infected lungs.

**Conclusions:**

These results provide strong evidence that the aberrant lung surfactant homeostasis in the LEC rats is caused by Rab^3^8 deficit, and suggest that endobronchial delivery of the responsive transgene could be an effective method to ameliorate the abnormal lung phenotype in the animal model of HPS.

## Background

Rab^3^8 small GTPase regulates intracellular transport of melanogenic substance in melanocytes [1, 2] and possibly lung surfactant in alveolar type II cells [3, 4]. Rab^3^8-deficient rats are a rat model of genetically heterogeneous Hermansky-Pudlak syndrome (HPS), which is clinically characterized by oculocutaneous albinism, bleeding diathesis, and in the majority of cases fatal interstitial pneumonia [5–7]. These rats include several Long Evans rat sub-strains carrying the phenotype of oculocutaneous albinism and bleeding diathesis. The mutation responsible for the phenotype (*Ruby*) was identified as a point mutation in the initiation codon of the Rab^3^8 small GTPase that resulted in the null translation of the protein [3, 7].

Previously, we reported altered lung surfactant system in Long Evans Cinnamon (LEC) rats, one strain of the *Ruby* rats [3]. The lungs exhibited type II cells with morphological changes characterized by remarkably enlarged lamellar bodies. Hydrophobic surfactant constituents were increased in lung tissues and lamellar bodies. Isolated LEC type II cells exhibited aberrant secretory patterns of newly synthesized [^3^H]phosphatidylcholine. Thus, Rab^3^8-deficient type II cells appeared to harbor abnormal lung surfactant secretion. Since some of these changes were also observed in *chocolate* mice carrying another Rab^3^8 mutation, Rab^3^8 mutation was considered to be responsible for the abnormal lung surfactant homeostasis [4, 8]. These lung pathological changes also shared significant similarities with those observed in human HPS lung tissues with unknown genetic background [9].

Since Rab^3^8 mutation appears to cause oculocutaneous albinism, bleeding diathesis, and lung abnormalities, it is expected that appropriate exogenous expression of Rab^3^8 in the lungs will restore ameliorated homeostasis of lung surfactant. However, Rab family protein requires several Rab regulator proteins to correctly function between GDP- and GTP-bound forms in the cell [10, 11]. Therefore, it is not clear whether exogenously expressed Rab^3^8 effectively functions until exogenous introduction of Rab^3^8 into the *Ruby* rat lungs result in improvement of the lung surfactant abnormalities. The goal of this study was to investigate whether adenovector-mediated endobronchial gene delivery of Rab^3^8 into the lungs could improve aberrant lung surfactant homeostasis in the LEC rats.

## Methods

### Reagents

Unless otherwise specified, chemicals were purchased from Sigma (St. Louis, MO) or Wako Chemicals (Osaka, Japan). Restriction enzymes were obtained from Nippongene (Tokyo, Japan). Rabbit anti-rat Rab^3^8 polyclonal antibody was produced in our laboratory [8]. Mouse anti-pig surfactant protein B (SP-B) monoclonal antibody (8B5E) was a generous gift from Dr. Yasuhiro Suzuki (Department of Molecular Pathology, Chest Disease Research Institute, Kyoto University), and mouse anti-rabbit glyceraldehyde-3-phosphate dehydrogenase (GAPDH) antibody was purchased from Chemicon (Temecula, CA). Mouse anti-rat surfactant protein A (SP-A) monoclonal antibody (1D6) was a generous gift from Dr. Dennis R. Voelker, the National Jewish Health (Denver, CO). Horseradish peroxidase-conjugated goat anti-rabbit IgG antibody, anti-mouse IgG antibody, and a broad-range prestained SDS-PAGE molecular marker were purchased from Bio-Rad (Hercules, CA). A chemiluminescent detection kit, a stripping buffer, and a micro bicinchoninic acid (BCA) protein assay kit were purchased from Pierce (Rockford, IL). A thin-layer chromatography apparatus was purchased from Advantec (Tokyo, Japan), and Silica gel G plates were purchased from Analtec (Uniplate, Newark, DE).

### Construction of recombinant adenovirus

**Fig. 1:**
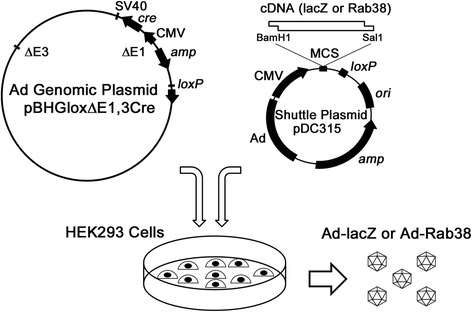
Construction of recombinant adenovirus. A multi-cloning site (MCS) of a shuttle plasmid pDC315 was digested with BamH1 and Sal1 restriction enzymes. cDNA (lacZ or Rab^3^8) was digested with the two enzymes and inserted into the shuttle plasmid. The recombinant shuttle plasmid (lacZ or Rab^3^8) and the adenovirus genomic plasmid were simultaneously added to HEK293 cells. Recombinant viral plaques appeared within 2–3 weeks. See the text for detail

An adenovector construction kit (AdMax) and HEK293 cells were purchased from Microbix Biosystems Inc. (Toronto, Ontario, Canada) [12] (Fig. 1). We digested a shuttle plasmid pDC315 from the kit with BamH1 and Sal1 enzymes to construct a recombinant plasmid containing lacZ-cDNA or Rab^3^8-cDNA. The lacZ-cDNA was excised from the pRSET/lacZ plasmid (Invitrogen, Carlsbad, CA) using BamH1 and Hind3 enzymes, inserted into a pBlueBacHis2A (Invitrogen) and then re-digested with BamH1 and Sal1 enzymes. Rab^3^8-cDNA was excised from pBlueBacHis2A-Rab^3^8 [8] using BamH1 and Sal1 enzymes. All plasmids, including an adenovirus genomic plasmid (pBHGloxΔE1,3Cre) which harbor cytomegalovirus (CMV) promoter and *Cre/loxP* recombination sites, were purified by CsCl-density gradient ultracentrifugation. HEK293 cells were co-transfected with either of the recombinant shuttle plasmids (lacZ or Rab^3^8) and the adenovirus genomic plasmid which lacked the early-transcribed regions (E1 and E3) according to the manufacturer’s protocol. The 293 cells harbor the early-transcribed regions (E1 and E3) for replication of the recombinant adenovirus. Recombinant viral plaques generated from Cre-lox recombination appeared within 2-3 weeks, some of which were isolated and propagated. The expression of lacZ and Rab^3^8 were confirmed by β-galactosidase enzyme (lacZ) assay on live 293 cells and Western blot analysis of 293 cell lysates with a rabbit anti-Rab^3^8 polyclonal antibody [8], respectively. The viruses were amplified using 293 cells, and purified high-titer adenovirus stocks were prepared with CsCl_2_-density gradient ultracentrifugation, subsequently dialyzed against 10 mM Tris-HCl, pH 8.0, aliquoted in presence of 10% glycerol, and stored at –80 °C.

### Rats

All animal protocols were reviewed and approved by the Institutional Animal Care and Use Committee of Kanazawa Medical University. Specific-pathogen-free (SPF) male Sprague-Dawley (SD), Long Evans/Kwl (LE), and Long Evans Cinnamon/Crj (LEC) rats were purchased from Japan Charles River Inc. (Yokohama, Japan) and housed in the SPF rat room of Kanazawa Medical University Laboratory Animal Center. The genotypes of the rats were determined by DNA sequencing of PCR products of Rab^3^8 exon 1 as previously reported [3]. Eight to twelve-week-old male rats, weighing 148–249 g, were used. For adenovector administration experiments, LE or LEC rats were anesthetized by intraperitoneal administration of pentobarbital sodium (~15 mg) and intubated with a 16-G plastic venous catheter. Through the catheter, an extra-thin soft polypropylene catheter connected to an adenovector-containing 1-ml syringe was inserted into the left lung, and purified high-titer adenovirus (10^9^ pfu in 0.5 ml PBS/1% glycerol) was slowly delivered into the left lung at the left decubitus position (inclined head-up), followed by several air injections to flush the airway tract [13]. At 2-weeks post administration, the rats were anesthetized with intraperitoneal injection of pentobarbital sodium (~ 15 mg) and sacrificed by cutting abdominal aorta, and used for further investigation.

### Isolation and culture of alveolar type II cells

Eight- to ten-week-old male SD or LEC rats were used. Type II cells were isolated from these rats by tissue dissociation using elastase and metrizamide density-gradient centrifugal separation as previously described [14–16]. Isolated cells were immediately suspended in DMEM/10% FBS with the recombinant adenovirus (Ad-lacZ or Ad-Rab^3^8) at a multiplicity of infection (MOI) (13) of 5 and incubated for 1 h in a 37 °C water bath with gentle shaking. Next, the cells were seeded at 2 × 10^6^ cells in 35-mm plastic culture dishes. The next day, adherent cells were washed, and fresh DMEM/10% FBS was replenished every 2 days.

### Fluorescence microscopy

Freshly isolated type II cells were plated on a plastic chamber slide (Lab-Tek II chamber slides, Nalge Nunc International, Naperville, IL). After overnight culture, the cells were infected with adenovirus (Ad-lacZ or Ad-Rab^3^8) at MOI = 2 to reduce expression level of Rab^3^8 protein and facilitate identification of co-localization with SP-B. The cells were further cultured for 24 h and washed twice with PBS. The cells were fixed with 4% paraformaldehyde for 10 min and followed with 100% acetone for 30 s. The fixed cells were blocked with 25% normal goat serum, incubated with a primary antibody (anti-Rab^3^8 and anti-SP-B), followed by a secondary antibody (Alexa 488-conjugated goat anti-rabbit IgG antibody and Alexa 594-conjugated goat anti-mouse IgG antibody) (Abcam, Tokyo, Japan). The slides were mounted with an antifade solution containing DAPI (S7113, Millipore, Temecula, CA). The immunofluorescence images were acquired using a fluorescence microscope (Keyence Biorevo BX-9000, Osaka, Japan).

### [^3^H]Phosphatidylcholine (PC) secretion

Male SD rats were used as controls, and male LEC rats were used as Rab^3^8-deficient rats. Type II cells were isolated as described above [14–16]. Isolated cells were infected with adenovirus (Ad-lacZ or Ad-Rab^3^8) at MOI = 5 for 1 h and plated in 35-mm plastic culture dishes at 2 × 10^6^ cells in 2 ml of DMEM containing 10% FBS and incubated in a 10% CO_2_ incubator at 37 °C for 22 − 24 h. The adherent cells were replenished with 2 ml of DMEM containing 10% FBS and 0.5 μCi/ml of [^3^H]choline chloride (ARC, St. Louis, MO) and further cultured for 22 − 24 h. After wash out of the [^3^H]-containing medium, 1.8 ml DMEM at 37 °C was added to the adherent cells and incubated for 15 min, followed by addition of the following agonist: 12-*O*-tetradecanoyl-phorbol-13-acetate (TPA, 100 nM), ATP (10 μM), and terbutaline (100 μM). Phospholipid secretion was allowed to proceed for 3 h. The supernatant media and cells were separated, and the lipids were extracted according to the Bligh-Dyer method [17], and [^3^H]phosphatidylcholine (PC) was counted using a liquid scintillator. Basal secretion values (%) were expressed as 100 × [^3^H]PC in supernatant/(supernatant + cell). Agonist-induced secretion value was normalized by the basal secretion value within each experimental group, i.e. fold of the basal secretion value. LDH release was evaluated using a LDH Cytotoxicity Detection Kit (Clontech, Mountain View, CA); less than 3% of LDH released into the media from total cellular LDH was considered as insignificant cellular toxicity.

### Preparation of bronchoalveolar lavage fluids, lamellar bodies, and lung homogenates

Eight- to twelve-week-old male LE or LEC rats were used. The left lungs were excised and weighed. Next, a 2.5 ml of saline/10 mM Hepes (pH 7.4) was infused into the left lung and gently recovered. This procedure was repeated 4 times, yielding approximately 12 ml of lavage fluid. The half volume of cell-free bronchoalveolar lavage (BAL) fluids were concentrated to a 0.5-ml volume with a centrifugal filter (MWCO 5000, Millipore). The remaining lung was weighed and cut into two parts of equal weight. One part was cut into small pieces and homogenized using a Potter-Elvehjem-type homogenizer. One-tenth volume was reserved as a lung homogenate, and the remaining nine-tenths were further used for lamellar body purification [15, 16]. Discontinuous sucrose density gradients were centrifuged at 100,000 × g for 3 h. The 0.4–0.6 M layers were recovered as the lamellar body fraction, diluted, and centrifuged at 100,000 × g for 1 h to pellet the lamellar bodies. The pellet was divided into two portions, which were used for Western blot and lipid extraction/phosphorus assay. The other half of the left lung was cut into small pieces and used for lipid extraction with Bligh-Dyer lipid extraction and subsequent phosphorus assay [17, 18].

### Western blot

Samples containing fixed amount of protein were subjected to 4–12% Bis-Tris SDS-PAGE under reducing condition and transferred to nitrocellulose membranes. The membranes were immunoblotted with a rabbit anti-rat Rab^3^8 antibody, or a rabbit (or mouse) anti-surfactant protein antibody, followed by incubation with a horseradish peroxidase-conjugated goat anti-rabbit (or anti-mouse) IgG antibody. Chemiluminescent detection assay was performed, and the bands were developed using an autofluorography film. Exposure times were adjusted to between 15 s and 5 min (usually 1 min) depending on the signal intensities obtained. When indicated, after stripping off the antigen-antibody complex, the same membrane was reused for the second antigen-antibody reaction.

### Phospholipid assay

Total lipids were extracted according to the Bligh and Dyer method [17] from one of the two cell-free BAL fluids, one of the two lamellar body fractions, and half of the left lung. In all cases, 20% of each sample was subjected to phospholipid phosphorus content assessment according to the method described by Bartlett [18]. The remaining 80% of each sample was subjected to two-dimensional thin-layer chromatography [19]. After development, the silica gel spots corresponding to each phosphatidylcholine species were scraped off and recovered quantitatively. The phosphorus content was then quantified by the Bartlett method [18].

### Electron microscopy

Two weeks after the administration of adenovirus, the LE or LEC rats were sacrificed, and freshly excised lungs were cut into small pieces and fixed for 2 h with fresh fixative containing 2.5% glutaraldehyde/0.1% picric acid/2% osmium tetroxide/4% sucrose/0.1 M cacodylate buffer (pH 7.4). The blocks were post-fixed with 1% aqueous uranyl acetate solution for 1 h followed by dehydration in a graded series of ethanol and subsequent propylene oxide. The samples were finally embedded in Epon. Semi-thin sections (0.8 μm-thick) were prepared and stained with 1% toluidine blue with warming. Ultrathin sections (60 nm-thick) were then counterstained with 2% uranyl acetate, followed by 2.6% lead nitrate/3.5% sodium citrate (pH 12). The sections were examined using a Hitachi H-7100 transmission electron microscope. Using ~25 electron microscopic photographs per experimental group, the areas of cells and lamellar bodies were quantified by an area-calculating software (Area Manager Lite, Visionary Co. Ltd., Kobe, Japan), and the numbers of lamellar bodies per single cell were counted.

### Statistics

Data were expressed as mean ± SEM. Single-factor ANOVA was used for data analysis among the three experimental groups and followed by Student-Newman-Keuls *post hoc* test for multiple comparisons. *P* values <0.05 was considered as statistically significant.

## Results

### The constructed recombinant adenovirus effectively expresses the enzyme of interest

**Fig. 2:**
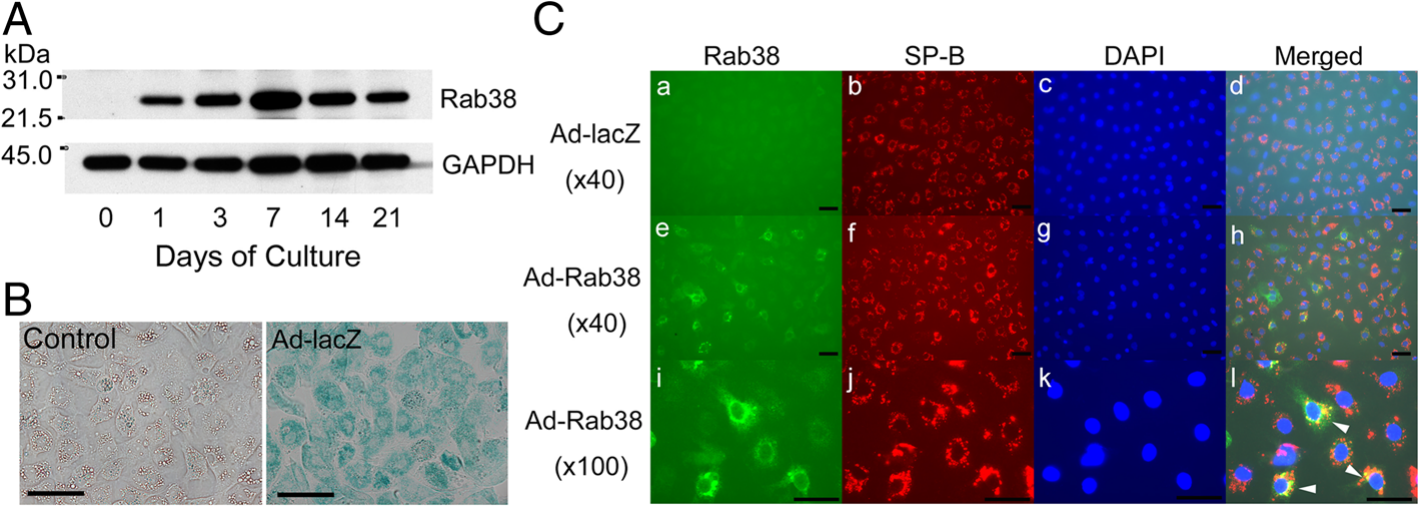
Expression of Rab^3^8 protein in LEC type II cells infected with Ad-Rab^3^8. (**a**) Western blot shows expression of Rab^3^8 protein by Ad-Rab^3^8 infection from day 1 through day 21. (**b**) The β-galactosidase (lacZ) assay shows ~100% of transduction efficiency of rat type II cells by Ad-lacZ infection at MOI = 5. Bar = 20 μm. (**c**) Immunofluorescence staining of exogenously expressed Rab^3^8 in LEC type II cells. Adherent cells were infected with Ad-lacZ (*a* – *d*) or Ad-Rab^3^8 (*e* – *l*). Objective lens magnification, *a* − *h*: ×40, i − l:×100. Bar = 25 μm. The exogenous Rab^3^8 appeared to at least partially co-localize with granularly distributed SP-B in the cells (*l*, arrow heads)

The adenovirus carrying lacZ-cDNA (Ad-lacZ) at MOI of 5 infected ~ 100% of cultured type II cells in vitro and expressed functional β-galactosidase (Fig. 2b). The Ad-lacZ-infected and -non-infected (control) cells were stained with 0.5 mg/ml X-gal. Western blot (Fig. 2a) confirmed exogenously expressed Rab^3^8 in Ad-Rab^3^8- infected LEC type II cells in primary culture. Freshly isolated cells (day 0) were infected with Ad-Rab^3^8 at MOI = 5 for 1 h and cultured for the indicated time. The post nuclear supernatant equivalent of 0.5 × 10^6^ cells was used for the analysis. As previously reported [3], intact LEC type II cells completely lacked Rab^3^8 protein. In contrast, Ad-Rab^3^8-infected cells expressed Rab^3^8 protein (molecular weight ~ 26 kDa) by 24 h after infection. This expression persisted for more than 21 days with a peak in expression at approximately 7 days. Immunofluorescence cell staining of cultured LEC type II cells with anti-Rab^3^8 antibody revealed no protein expression in intact LEC type II cells (Fig. 2c; a), but significant exogenous Rab^3^8 protein in Ad-Rab^3^8-infected LEC type II cells (Fig. 2c; e, i). The exogenous Rab^3^8 appeared to at least partially co-localize with granularly distributed SP-B in the cells (Fig. 2c; l, arrow heads).

### Effect of exogenously expressed Rab^3^8 on surfactant phosphatidylcholine secretion from Rab^3^8^-/-^ alveolar type II cells

**Fig. 3:**
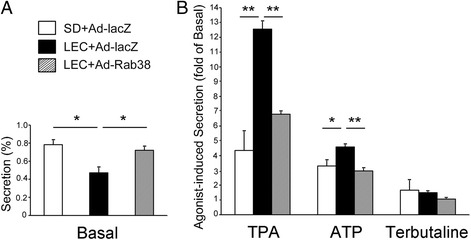
[^3^H]phosphatidylcholine secretion from cultured type II cells isolated from SD or LEC rats. The cells were infected with Ad-lacZ or Ad-Rab^3^8 and radiolabeled with [^3^H]choline. Secretion (%) was calculated as [^3^H]PC activity in 100 × supernatant/(supernatant + cell). **a** Basal secretion (%) was without an agonist. **b** Agonist-induced secretion value was normalized by basal secretion value in each experiment group, i.e. fold of the basal secretion value. *P < 0.05, **P < 0.01. *n* = 6 of separate experiments performed in duplicate dishes

Type II cells are unique cells that specifically synthesize large amount of [^3^H]PC as a predominant surfactant constituent using [^3^H]choline chloride precursor that is added to the culture medium. [^3^H]PC is hydrophobic and is extracted into organic phase, whereas [^3^H]choline chloride is hydrophilic and extracted into hydrophilic phase using the Bligh-Dyer lipid extraction method [17]. Figure 3 shows the secretion of newly synthesized [^3^H]PC from cultured type II cells. Compared with wild-type (SD) cells, basal secretion is significantly reduced in Ad-lacZ-infected LEC cells (Fig. 3a). Basal secretion in Ad-Rab^3^8 LEC cells was significantly higher than Ad-lacZ-infected LEC cells, which exhibited no significant difference compared with the wild-type cells. TPA, ATP, and terbutaline are known as an agonist for PC secretion from cultured type II cells [14, 20]. TPA- and ATP-induced [^3^H]PC secretion was remarkably increased in LEC cells as previously reported [3] (Fig. 3b). However, TPA- and ATP-induced [^3^H]PC secretion from Ad-Rab^3^8-infected LEC cells was significantly decreased compared to that from Ad-lacZ-infected LEC cells. Basal, TPA-, ATP-, and terbutaline-induced [^3^H]PC secretion from Ad-Rab^3^8-infected cells were not significantly different from that of wild-type cells.

### Transduction efficiency by a single endobronchial administration of adenovector

**Fig. 4:**
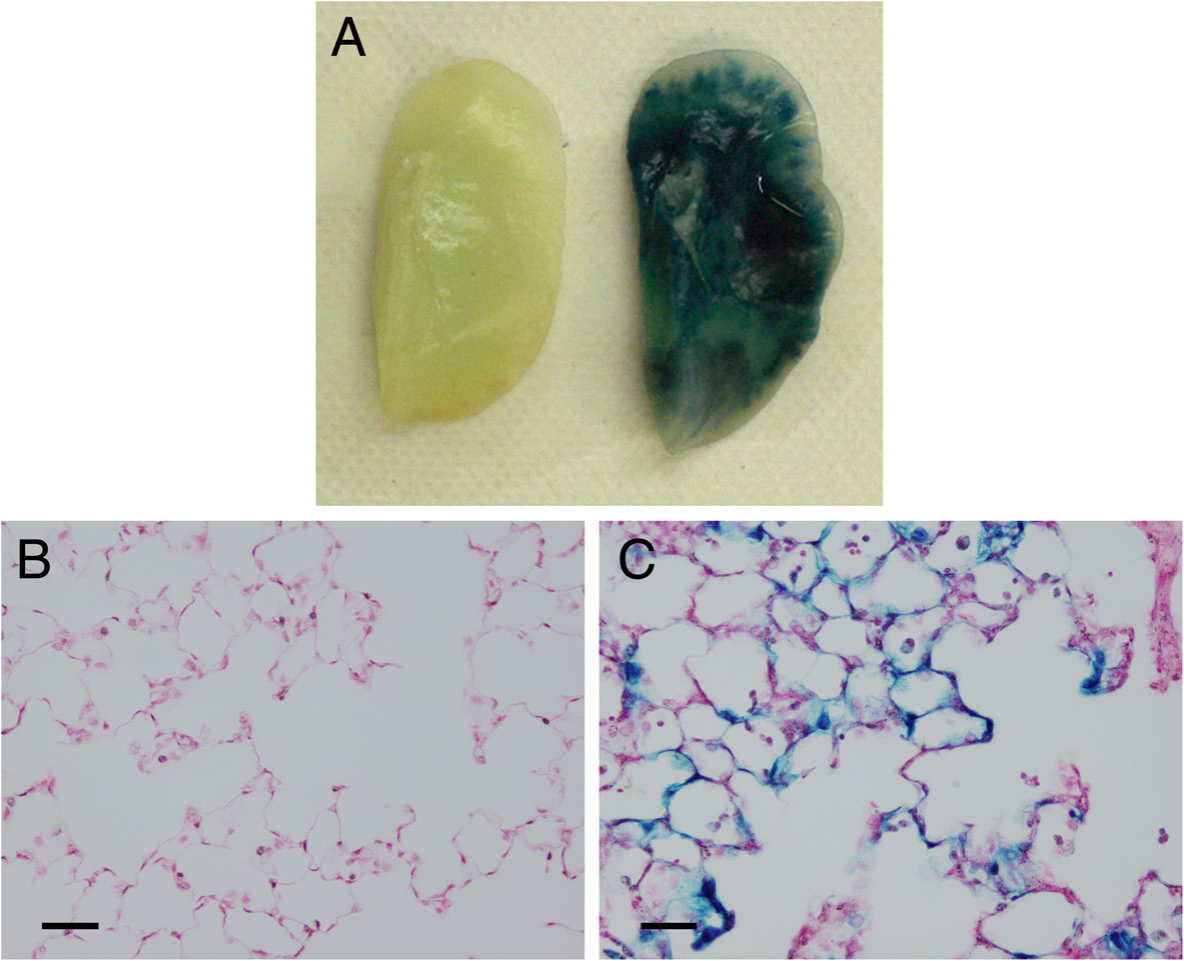
Transduction efficiency by a single endobronchial administration of Ad-lacZ as evaluated by ex vivo endobronchial staining with lacZ assays. **a** Ad-lacZ was delivered into the left lungs by a single endobronchial administration. Fourteen days later, the lungs were stained for 4 h with intratracheal administration of 0.5 mg/ml X-gal (13). *Left*: Control, *Right*: Ad-lacZ. **b**, **c** The lung tissues were sectioned into 5-μm slices, and counter-stained with nuclear fast red (Kernechtrot). Magnification × 200, Bar = 40 μm. **b**: Control, **c**: Ad-lacZ

The efficiency of adenovector (Ad-lacZ) spreading into the left lung by a single endobronchial instillation was evaluated by X-gal staining of a whole left lung. Two-week post-administration of Ad-lacZ or vehicle (0.85% NaCl/1.0% glycerol), the left lungs were excised, fixed, and stained for 4 h with an intratracheal administration of a 0.5 mg/ml of X-gal/2 mM MgCl_2_/5 mM K_4_Fe(CN)_6_/5 mM K_3_Fe(CN)_6_/20 mM Tris/PBS (pH 8.3) [13]. The Ad-lacZ-administered lung (Fig. 4a, right) exhibited extensive staining by a lacZ assay, suggesting the effectiveness of the administration. The stained lung was embedded in paraffin, sectioned, and counter-stained with nuclear fast red (Kernechtrot) stain. The lower respiratory tract including alveolar tissues also exhibited effective staining by the lacZ assay (Fig. 4c). These results indicate that a single endobronchial administration of adenovector can effectively transduce lower respiratory tract cells.

### Effect of an endobronchial administration of Ad-Rab^3^8 on lung surfactant homeostasis in Rab^3^8^-/-^ rat lungs

**Fig. 5:**
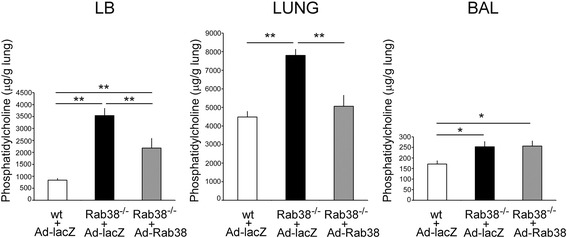
Phosphatidylcholine levels in Ad-Rab^3^8-infected LEC rat lungs. Ad-lacZ or Ad-Rab^3^8 recombinant adenovector was delivered into the left lungs by an endobronchial administration at 14 days prior to sacrifice. The left lungs were lavaged, homogenized, and lamellar body (LB) fractions were isolated. After lipid extraction, phosphatidylcholine levels were analyzed by two-dimensional thin layer chromatography. *P < 0.05, **P < 0.01 (*n* = 6 rats). Note the different magnitude of a vertical scale

Two-week post-administration, the left lungs were weighed and lavaged with a saline/10 mM Hepes (pH 7.4). The remaining lungs were homogenized, and lamellar body fractions were isolated by sucrose density-gradient ultra-centrifugation. Total lipid contents were extracted from cell-free bronchoalveolar lavage fluids, lamellar body fractions, and lung homogenates. Phosphatidylcholine was analyzed by two-dimensional thin layer chromatography. Phosphatidylcholine levels in the lamellar body fractions and lung homogenates in Ad-Rab^3^8-infected lungs were significantly lower than those in Ad-lacZ-infected lungs (Fig. 5). However, phosphatidylcholine levels in the bronchoalveolar lavage fluids did not differ.

**Fig. 6:**
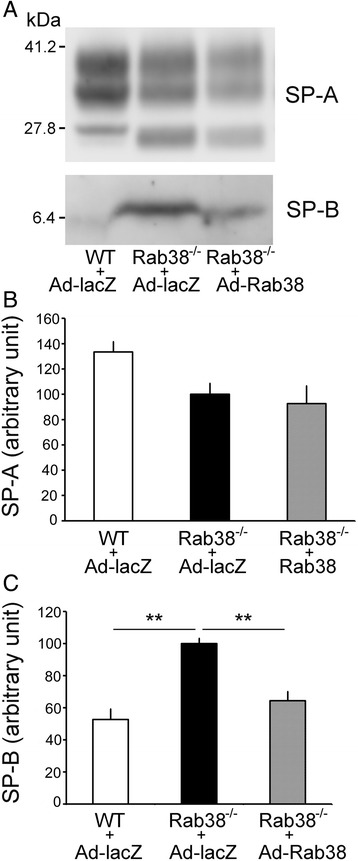
Surfactant protein B is decreased in the lamellar body fraction in Ad-Rab^3^8-infected LEC rat lungs, whereas surfactant protein A is not altered. Fixed amounts of the lamellar body fractions (5 μg protein) were loaded onto SDS-PAGE and transferred to a nitrocellulose membrane. The same membrane was incubated twice with two different primary antibodies after being stripped of previous antibody complexes. **a** A representative result from three independent experiments, each with similar results. Densitometry of the Western blots was performed for SP-A (**b**) and SP-B (**c**). **P < 0.01, *n* = 3 rats

The SP-A and SP-B amounts in the lamellar body fractions were evaluated by Western blot (Fig. 6a). Fixed amounts of the resuspended lamellar body fraction (5 μg protein) were used (*n* = 3 rats for each group). Densitometry indicated that SP-B was significantly decreased in Ad-Rab^3^8-infected lungs compared to Ad-lacZ-infected lungs (Fig. 6c), whereas SP-A did not differ (Fig. 6b).

### Effect of an endobronchial administration of Ad-Rab^3^8 on morphology of alveolar type II cells and their lamellar bodies in Rab^3^8^-/-^ rat lungs

**Fig. 7:**
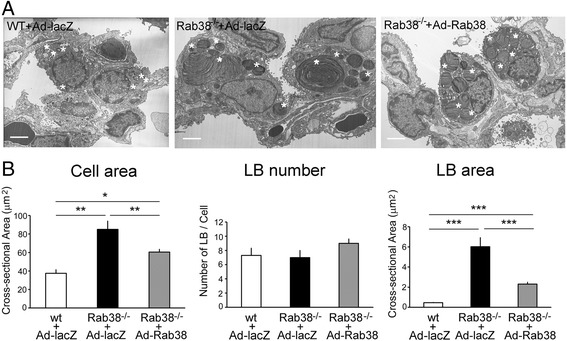
Electron microscopic appearance of type II cells and lamellar bodies in Ad-Rab^3^8-infected LEC rat lungs. **a** Ultra-thin sections (60 nm-thick) prepared from the left lungs were examined using a transmission electron microscope, and more than 25 fields were randomly photographed at a magnification of × 6,000. Star (*) indicates lamellar body. Bar: 6.6 μm. **b** Using ~25 electron microscopic photographs per experimental group, the areas of cells and lamellar bodies were quantified by an area-calculating software, and the numbers of lamellar bodies per single cell were counted. *P < 0.05, **P < 0.01, ***P < 0.001

Compared with Ad-lacZ-infected wild-type (LE) lungs, type II cells in Ad-lacZ-infected LEC lung exhibited strikingly large lamellar bodies similar to intact LEC lungs as previously reported (Fig. 7a) [3]. In contrast, type II cells in Ad-Rab^3^8-infected LEC lung exhibited smaller lamellar bodies compared with Ad-lacZ-infected LEC lungs. Quantitative area measurement revealed that type II cells and lamellar bodies in Ad-Rab^3^8-infected LEC lungs were smaller than those of Ad-lacZ-infected LEC lungs, although they were still significantly larger than those of Ad-lacZ-infected wild-type lungs (Fig. 7b).

## Discussion

Previously, we observed alterations in surfactant metabolism in Rab^3^8-deficient LEC rats [3]. Isolated type II cells exhibited aberrant secretory patterns of newly synthesized [^3^H]PC, as indicated by decreased basal secretion and remarkably amplified agonist-induced secretion. [^3^H]PC synthesis and uptake by type II cells were not altered. The expression levels of mRNA for surfactant proteins A, B, C, and D were not altered. The LEC lungs contained type II cells with significantly altered morphology characterized by remarkably enlarged lamellar bodies. Surfactant phosphatidylcholine and surfactant protein B were increased in lung tissues and lamellar bodies. These results shared significant similarities with the lung histopathology in human HPS patients characterized by peculiar giant lamellar bodies in type II cells and lung phospholipidosis [9]. Rab^3^8 is highly expressed in alveolar type II cells [8, 21] as well as melanocytes and platelets [1, 22]. It is possible that Rab^3^8 deficiency cause the HPS lung phenotype, which is closely related to abnormalities in type II cells and their lung surfactant metabolism [9].

In this study, we observed that cross-sectional areas of cells and giant lamellar bodies in LEC rats were reduced close to wild-type rats at 2 weeks after Ad-Rab^3^8 delivery (Fig. 7). This result is similar to a recent report that enlarged lamellar body phenotypes were rescued by transient expression of EGFP-Rab^3^8 in cultured Rab^3^8-deficient rat type II cells in vitro [23]. Consistent with the cellular and lamellar body changes, lung surfactant PC pools were improved after the administration of Ad-Rab^3^8 (Fig. 5). PC levels were decreased in lung tissues and lamellar body fractions but were not altered in BAL fluid samples. SP-B levels evaluated by Western blot analysis were decreased within the lamellar body fraction, whereas SP-A levels were not altered (Fig. 6). Thus, the homeostasis of hydrophobic surfactant constituents (i.e., PC and SP-B) was ameliorated by the administration of Ad-Rab^3^8 into the lung.

Lung surfactant is a complex of several lipids (predominantly PC) and four surfactant apoproteins; SP-A, -B, -C, and -D [24]. PC, SP-B, and SP-C are hydrophobic and function as surface tension-lowering molecules, whereas SP-A and SP-D are minimally related to surfactant function but are closely related to innate immune function [25]. Growing evidence implicates different intracellular transport pathways for each surfactant component. With exception of SP-D, these surfactant components are stored within lamellar bodies [26]. SP-D is synthesized, transported to the Golgi apparatus, and then constitutively secreted; it is not routed to the lamellar bodies [27]. Newly synthesized SP-A is transported to the Golgi apparatus, undergoes glycosylation, and is then constitutively secreted [16]. A certain amount of secreted SP-A is subsequently transported into the lamellar bodies [16]. However, SP-A is not enriched in the lamellar bodies in the same manner as PC, SP-B, or SP-C [26]. Phosphatidylcholine, SP-B, and possibly SP-C are transported to the lamellar bodies, stored, and undergo regulated secretion [25, 27]. This study showed that Ad-Rab^3^8 did not affect the SP-A levels within the lamellar body fraction, whereas it ameliorated PC and SP-B levels, suggesting that Rab^3^8 specifically participates in the intracellular trafficking of PC and SP-B but not of SP-A in type II cells.

Hermansky-Pudlak syndrome (HPS) comprises a group of related autosomal recessive diseases that are genetically heterogeneous [5, 6, 28]. In human HPS, there are several responsive genes to cause HPS phenotype, including *HPS-1* to *-10* so far. In addition to oculocutaneous albinism and bleeding diathesis, the majority number of HPS-1, HPS-2, and HPS-4 patients suffer from life-threatening interstitial pneumonia without any effective therapeutic option [5, 6, 9]. The lung involvement in HPS patients is characterized by usual interstitial pneumonia (UIP)-like interstitial pneumonia [6, 9]. The prominent pathological features of the lung tissues are alveolar septa displaying florid proliferation of type II cells with characteristic foamy swelling/degeneration [9]. Those peculiar type II cells observed in HPS patient lungs are histochemically characterized by the over accumulation of phospholipids, and ultra-structurally by the presence of numerous giant lamellar bodies, suggesting a form of cellular degeneration associated with an over accumulation of surfactant (giant lamellar body degeneration). These results suggest the presence of a disorder of lung surfactant metabolism in HPS type II cells and that aberrant lung surfactant homeostasis might be involved in the pathogenesis of interstitial pneumonia.

There are 15 mouse homologues of human HPS that manifest oculocutaneous albinism and bleeding diathesis [29, 30]. Among them, ten genetically distinct forms of human HPS have been identified in humans (*HPS1–10*). In contrast, only the Rab^3^8-deficient rat (*Ruby*) has been recognized as a rat model of HPS [7, 31]. The *Ruby* mutation occurs in the initiation codon of Rab^3^8 exon1, which presumably results in a protein translation defect [7], as shown in Fig. 2. Experimentally engineered double mutant mice (*pale ear/pearl*, i.e., *Hps1/Hps2*) are used as mouse models of HPS lung pathology and exhibit lung inflammation and emphysema with prominent overloading of phospholipids in remarkably enlarged lamellar bodies [32]. These changes are strikingly amplified but share significant similarity with both *chocolate* mice [4], which harbor another Rab^3^8 mutation, and *Ruby* rats [3].

Most of the genetic products identified in the variant forms of HPS participate in vesicular trafficking that is related to lysosome-related organelles (LROs), as they are involved in the biogenesis of lysosome-related organelle complexes (BLOC) −1, −2, and −3 [28, 29, 33]. HPS-1 and HPS-4 patients develop fatal interstitial pneumonia at their third or fifth decade. The two causative genes encode BLOC-3 subunits, which have been elucidated to function as guanine nucleotide exchange factors (GEFs) for Rab^3^8 and its close homologue Rab^3^2 [11]. Silencing of the BLOC-3 subunits Hps1 and Hps4 results in the mislocalization of Rab^3^2 and Rab^3^8 and a reduction in pigmentation in a melanoma cell line [11]. In contrast, the molecular mechanism that Rab^3^8 deficiency causes perturbation of lung surfactant homeostasis in type II cells is largely unknown. However, the studies of other cell types such as melanocytes and megakaryocytes have provided insights on this mechanism. In these cells, the dysfunction of Rab^3^8 and its counterpart Rab^3^2 results in either failure of the trafficking of integral membrane proteins to mature melanosomes in melanocytes [10, 34] or of the fusion of immature cargo vesicles with mature vesicles (dense granules) in megakaryocytes [35]. Both melanosomes and dense granules are cellular organelles that are closely related to the endocytic pathway and lysosomes, and are hence lysosome-related organelles (LROs) [10]. Lamellar bodies and their closely related organelles, multivesicular bodies (MVB), are also LROs and participate in lung surfactant transport, secretion, and recycling [16, 20, 36, 37]. It is possible that Rab^3^8 is closely related to biogenesis of these LROs in type II cells.

## Conclusions

Adenovector-mediated gene transfer of Rab^3^8 effectively ameliorates lung surfactant secretion from isolated rat type II cells and aberrant lung surfactant homeostasis in the Rab^3^8-deficient rats. Our results support the direct role of Rab^3^8 in lung surfactant homeostasis in the animal model of HPS, and suggest that endobronchial delivery of the responsive transgene could be an effective method to ameliorate the abnormal lung phenotype in the animal model of HPS.

## Abbreviations

Ad-lacZ: Adenovector carrying lacZ-cDNA
Ad-Rab^3^8: Adenovector carrying Rab^3^8-cDNA
GAPDH: Glyceraldehyde-3-phosphate dehydrogenase
HPS: Hermansky-Pudlak syndrome
LEC: Long Evans Cinnamon
MOI: Multiplicity of infection
PC: Phosphatidylcholine
SD: Sprague-Dawley
